# Organs on chip approach: a tool to evaluate cancer -immune cells interactions

**DOI:** 10.1038/s41598-017-13070-3

**Published:** 2017-10-06

**Authors:** Elena Biselli, Elena Agliari, Adriano Barra, Francesca Romana Bertani, Annamaria Gerardino, Adele De Ninno, Arianna Mencattini, Davide Di Giuseppe, Fabrizio Mattei, Giovanna Schiavoni, Valeria Lucarini, Erika Vacchelli, Guido Kroemer, Corrado Di Natale, Eugenio Martinelli, Luca Businaro

**Affiliations:** 1 Department of Physics, Technische Universität München and Graduate School of Quantitative Biosciences, Ludwig-Maximilians-Universität München, James-Franck-Str. 1, 85748 Garching, Germany; 2 Dipartimento di Matematica, Sapienza Università di Roma, P.le A. Moro 5, 00185 Rome, Italy; 3 Dipartimento di Matematica e Fisica, Università del Salento, Via per Artesano, 73100 Lecce, Italy; 4 Institute for Photonics and Nanotechnologies, Italian National Research Council, Via Cineto Romano 42, 00156 Rome, Italy; 5 Department of Civil Engineering and Computer Science, University of Rome Tor Vergata, 00133 Rome, Italy; 6 Department of Electronic Engineering, University of Rome Tor Vergata, Via del Politecnico, 00133 Rome, Italy; 7 Istituto Superiore di Sanità—Department of Oncology and Molecular Medicine, Viale Regina Elena 299, 00161 Rome, Italy; 8 Metabolomics and Cell Biology Platforms, Gustave Roussy Cancer Campus, Villejuif, France; 9 Université Paris Descartes, Sorbonne Paris Cité, Paris, France; 10 Equipe 11 labellisée Ligue Nationale contre le Cancer, Centre de Recherche des Cordeliers, Paris, France; 11 Institut National de la Santé et de la Recherche Médicale, U1138 Paris, France; 12 Université Pierre et Marie Curie, Paris, France; 13 Pôle de Biologie, Hôpital Européen Georges Pompidou, AP-HP, Paris, France; 14 Department of Women’s and Children’s Health, Karolinska University Hospital, 7176 Stockholm, Sweden

## Abstract

In this paper we discuss the applicability of numerical descriptors and statistical physics concepts to characterize complex biological systems observed at microscopic level through organ on chip approach. To this end, we employ data collected on a microfluidic platform in which leukocytes can move through suitably built channels toward their target. Leukocyte behavior is recorded by standard time lapse imaging. In particular, we analyze three groups of human peripheral blood mononuclear cells (PBMC): heterozygous mutants (in which only one copy of the FPR1 gene is normal), homozygous mutants (in which both alleles encoding FPR1 are loss-of-function variants) and cells from ‘wild type’ donors (with normal expression of FPR1). We characterize the migration of these cells providing a quantitative confirmation of the essential role of FPR1 in cancer chemotherapy response. Indeed wild type PBMC perform biased random walks toward chemotherapy-treated cancer cells establishing persistent interactions with them. Conversely, heterozygous mutants present a weaker bias in their motion and homozygous mutants perform rather uncorrelated random walks, both failing to engage with their targets. We next focus on wild type cells and study the interactions of leukocytes with cancerous cells developing a novel heuristic procedure, inspired by Lyapunov stability in dynamical systems.

## Introduction

The quest to realize reliable experimental models to measure phenomena occurring in complex biological systems has become one of the frontiers of microfluidics. The idea of reconstituting the interactions among different cell populations or subsets of organ functionalities on small, microscopy-compliant, low cost, plastic devices is today a reality with concrete industrial applications and is known as the *organs-on-chip* (OOC) approach. These models allow the direct simultaneous observation of hundreds of different cells, moving, interacting and responding to signals emanating from the micro-environment nearby, thus giving access to numerous parameters describing the system as a whole that must be properly measured and elaborated. In the past years the combined efforts of our groups led us to set up a reliable model to study the interactions in the cancer-immune system cross-talk in defined scenarios including anticancer chemotherapy^1–4^. Empirically, it became clear that such complex systems can only be accurately described by novel approaches to deliver numerical descriptors of the biological system under study.

In a recent paper^5^ we introduced the idea of characterizing the dynamics of immune cells inside microfluidic devices in terms of a sharp set of numerical quantitative descriptors. In this paper we start from the main results presented there, which were based on experiments carried on a murine model, and extend them both in terms of application on human cells and of introducing new descriptors.

**Figure 1:**
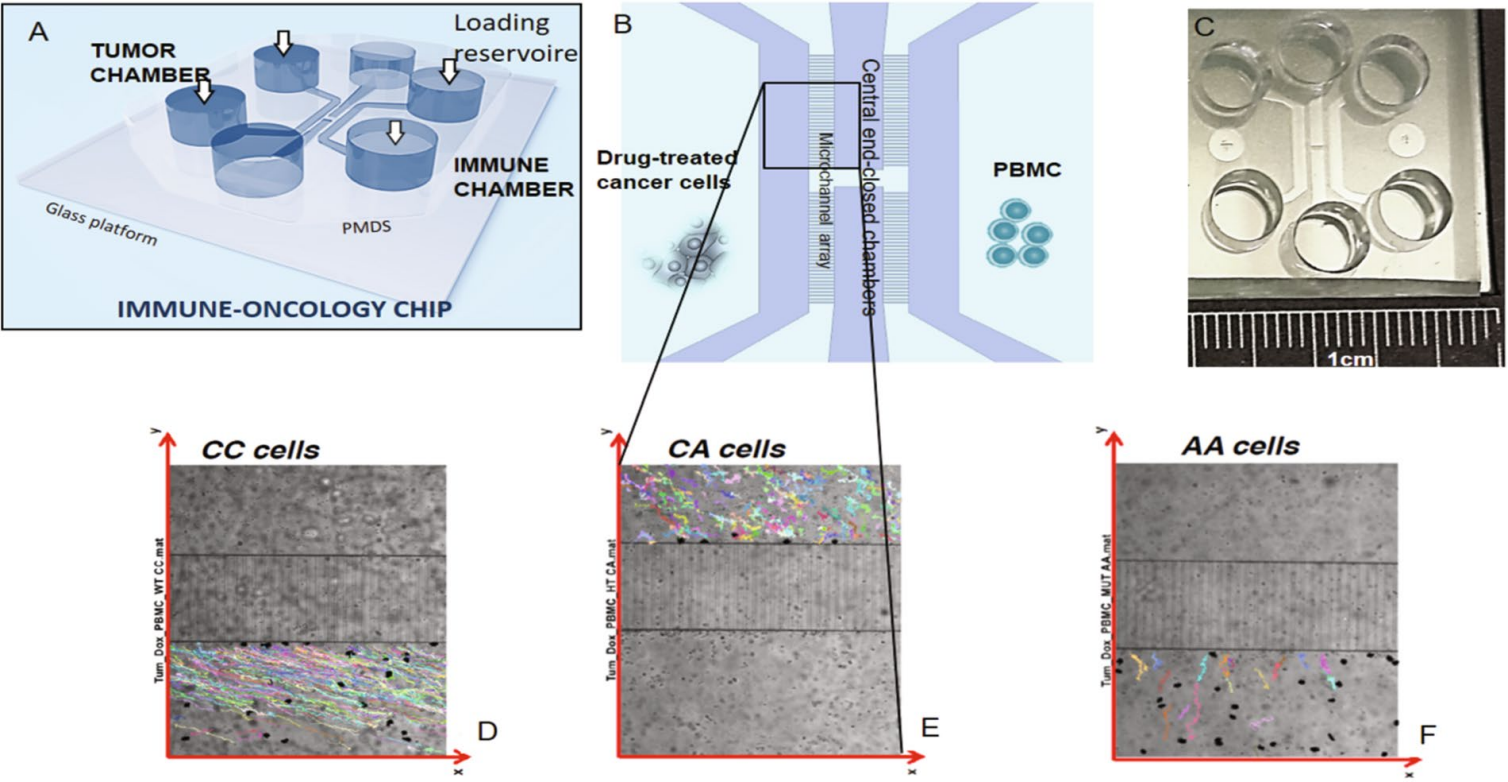
The immune-oncology chip hosting the experiments and track examples. Panel A) shows a general scheme of the device, composed by six reservoirs for cell loading and culture medium replacement and four chambers (or compartments) for cell culture. Panel B) presents a detailed view of the four chambers. The left chamber is dedicated to TCs culture, while PBMC, initially loaded in the right chamber, passively move in the two central chambers where they stop until sensing the chemo-attractant signals from treated TCs, thus starting to migrate towards them through the micro-channels array connecting the two culture chambers. A picture of the whole device is presented in panel C). In the lower part of the figure, we show examples of the typical responses performed by PBMCs in the three different settings. In each panel the reference system has the origin on the bottom left. The same reference system will be used throughout the data analysis. Panel D) shows the trajectories of pure FPR1 CC cells, able to migrate towards cancerous cells, which are loaded in the reservoir (not shown) at the bottom right of the channel; panel E) reports the trajectories of FPR1 CA cells, characterized by a weaker drift in the motion towards the target; panel F) shows the prevalently erratic behaviors of FPR1 AA cells. In all the three cases the black spots represent TCs.

More in detail, we apply this non-conventional analysis to the data obtained in a set of experiments described in one of our recent papers^1^. The rationale of the experiments was to study the interaction between human cancer cells (breast and colon), which were treated with chemotherapeutic agents, and human peripheral blood mononuclear cells (PBMC), which carried different genetic variants of the FPR1 gene. This gene codes for a 7 transmembrane G-protein-coupled receptor, formyl peptide receptor 1 (FPR1) that senses a ligand emanating from dying cancer cells, annexin A1. A loss-of-function allele of FPR1 can be present in individuals either in a heterozygous way (meaning that one allele of FPR1 is normal and the other dysfunctional) or in a homozygous fashion (meaning that both alleles of FPR1 are inactive). At clinical level the data collected correlated with the fact that patients that were heterozygous carriers of the FPR1 loss-of-function allele manifested a poor prognosis after anthracycline-based breast cancer chemotherapy. Similarly, colon cancer patients that were homozygous for the FPR1 loss-of-function allele failed to respond to oxaliplatin-based chemotherapy^1^. The experiments analyzed in this paper were performed in microfluidic platforms (see Fig. 1) and show the interaction between breast cancer cells and PBMC cells obtained from healthy donors bearing the FPR1 allele in homozygosis (CC), the RS867228 loss of function allele of FPR1 in heterozygosis (CA) and the RS867228 loss of function allele of FPR1 in homozygosis (AA).

The common underlying feature resulting from our OOC architecture is that tumor cells (TC) and PBMC immune cells can be placed in different compartments of the Immuno-Oncology chip so that they can move from one compartment to another via dedicated microchannels (see Fig. 1). This allows the observation of the entire PBMCs migratory behavior and, via standard time lapse imaging, to collect all their trajectories and to record the overall evolution of the system in its phase space. In this way we can address the cross-talk between human PBMCs and breast TCs and demonstrate that, for a successful chemotherapy in breast cancer, a competent antitumor immunity plays a crucial role in promoting interactions between PBMC and cancer cells^1^. In particular, FPR1-expressing PBMCs must firmly bind its TC target: this result highlights the necessity of a protocol able to quantitatively predict how many PBMCs (among the whole migratory PBMCs-flux) effectively collapse on their TC targets. In order to address this task, we present a novel approach based on statistical mechanics^6–17^ and dynamical systems analysis^18–21^. Adapting classical stability analysis, we study immune cell’s convergence to stable fixed points where doxorubicin-treated apoptotic cancer cells lie. We define TCs as *attractors* and locally prevailing source of the chemotactic gradients driving the PBMC and we show that, despite lacking the explicit equation of motion for PBMCs migrations, we can still treat their trajectories under a linear approximation, when PBMCs are in the neighborhood of the TCs: system’s dynamics can be locally described by a set of linear equations, whose Jacobian matrix can be trivially diagonalized in order to study its eigenvalues. Inspection of the latter allows a qualitative description of the environment: if both eigenvalues are negative, the PBMCs are going to engage a stable interaction with the TC (i.e., the motion gets stack in a minimum of the chemoattractive potential landscape associated to the TC itself); if their signs disagree, the PBMCs are partly attracted by the TC but not as much as required to steadily remain around it (the motion gets on a *saddle*); finally, if both the eigenvalues are positive, the PBMCs would be repelled by the tumor cell and move away: checking these possible outcomes in our OOC experiment is thus a routine that we perform extensively over the whole data-set, where we find robust evidence for attractive and partly attractive behaviors. The procedure is therefore proposed to provide an additional tool for a quantitative description of the immune response to cancer during chemotherapy. Moreover, this analysis could allow to reconstruct the effective *chemoattractant potentials* generated by the tumor cells: once the stability analysis has been performed over all the TCs involved in the experiment, we can infer their effective *local equilibrium* and overlap them in the physical space deriving the corresponding characteristics from the dynamic parameters extracted from time lapse data.

## Results

The results presented hereafter are divided in two different logical steps. The first section shows that the main quantitative descriptors presented in^5^ can be successfully used to classify a human coculture model too. In the second section, we extend the analysis applying dynamical stability criteria^22–24^ to the trajectories of human PBMCs moving toward single TCs, considered as attractors and locally prevailing sources of the chemotactic gradients driving the PBMCs. In particular, once justified experimentally that (close to attractors) the motion is locally linear, we show that Lyapunov coefficients can be reliable descriptors of the efficacy of motion and interaction of PBMCs and TCs. Our heuristic adaptation of stability analysis can thus determine when and how the convergence of the PBMC toward their target(s) in TCs can be referred to as stable, that is ultimately a necessary criterion for a proper immune response^1^. Finally it is worth stressing that our method reverses the classical perspective of stability theory: instead of inspecting properties of the immune cells, whose analysis is carried on considering the surroundings as known (usually in form of potential), we get information on the latter studying how immune cells approach tumor cells. Hence, immune cells work as *probes* to explore their surroundings and we aim to reconstruct the chemo-attractive landscape that tumor cells generate (i.e. the surrounding) by inspecting how PBMCs *walk* in this landscape.

### Distributional analysis of immune cells migration

In this section we analyze the motion of human PBMCs cultured in the microfluidic platform described in the Introduction and in Sec. 3, looking at the migratory ability of CC, CA and AA cells. Results showed that FPR1-deficient (KO) PBMCs (CA and AA) failed, at different levels depending whether the mutation of FPR1 occurs in heterozygosis or homozygous, to interact with treated cancer cells with respect to normal PBMCs (CC). The cell trajectories, identified as described in Sec. 4, could present a directional (bias) component induced by the gradient of chemokines produced by the overall ensemble of TCs and a Brownian (stochastic) component. The degree of biased or stochastic motion, in each position, correlates with the degree of sensing ability of the cell under examination. Hence, we can model such paths using random walks characterized by discrete time steps (this follows naturally from the time lapse recording) and moving on a continuous two-dimensional Euclidean space. Indeed, in order to properly describe such motion, we have to build a Cartesian framework based on a bidimensional coordinate system, where (*x*,*y*) denotes the position of a cell. We can also unequivocally map the pair (*x*,*y*) into the pair (r,*θ*) representing the location of the cell in polar coordinates where *r* is the distance of the cell from the origin of the framework and *θ* is the polar angle. Now, being (*x*,*y*) and $(x^{\prime} ,y^{\prime} )$ two successive positions of an arbitrary cell, we denote with ${\rm{\Delta }}x=x-x^{\prime}$ and with ${\rm{\Delta }}y=y-y^{\prime}$ the distance covered by the cell (along the *x* and the *y* axis, respectively) during the time step considered (Δ*t* = 2 min).

**Figure 2:**
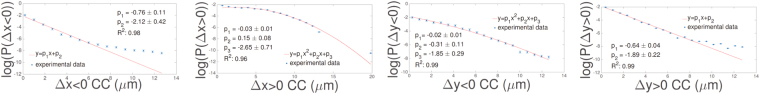
Distributions of step length for CC cells. For each CC cell we evaluate the extent of the motion performed during the time lapse between two consecutive measurements. This can be quantified in terms of displacement along the *x* and the *y* direction and the related step lengths are referred to as Δ*x* and Δ*y*. By merging experimental data from the whole set of tracks we build the distributions *P*(Δ*x*) and *P*(Δ*y*) shown in these panels in a semi-logarithmic scale, where experimental data are reported with standard errors. Neglecting the tails of the distributions (for which the statistics is rather poor), we can fit these distributions according to exponential and Gaussian functions, as reported in the panels together with the related fit coefficients. The motion resulting from these distributions displays a drift along positive *x* and negative *y*.

As previously demonstrated^5^, the proper characterization of the PBMC migration passes through the introduction and measurement of the distributions of step length along the two directions, namely *P*(Δ*x*) and *P*(Δ*y*). The mathematical form of the distribution that we obtain from the data fitting gives information about the kind of random walk that can model these paths at best (see Section S1 in supplemental material). Hence, according to the shape of the distribution, we can infer the effect of the chemokine’s gradient. In particular, qualitative differences emerge among the distributions pertaining to CC, CA, and AA cells (see Fig. 2 and Figs S1–S2 in supplemental material).

For CC cells the distribution of step length is exponential along negative *x* and positive *y*, while it is Gaussian along positive *x* and negative *y* (see Fig. 2), suggesting the existence of a drift which biases the motion along positive *x* and negative *y*, where, in fact, the tumor reservoir is placed (see Fig. 1). For CA cells the distribution of step length along the *x* direction follows the same behavior of that just described for CC cells (see Fig. S1). On the other hand, for the motion along the *y* direction, step lengths are distributed exponentially regardless of their sign. This indicates that the drift is weaker than the CC case and it acts mainly along the *x* direction. For AA cells the distributions of step length Δ*x* and of step length Δ*y* are always exponential regardless of their sign (see Fig. S2), suggesting that the bias is absent or too weak for cells to experience the effect of the chemokine’s gradient.

**Figure 3:**
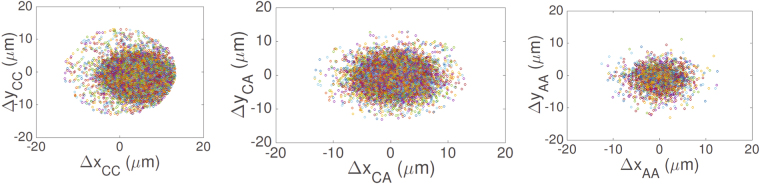
Scatter plots for step length along different directions. For each panel, each data point represents the step length along *x* and *y* direction performed by a cell during the time lapse between two consecutive measurements. Left panel: CC cells; central panel: CA cells; right panel: AA cells. Note that the clouds of data lack any internal structure of polarization.

The behaviors highlighted can be corroborated by considering the scatter plots presenting Δ*y* versus Δ*x* data (see Fig. 3): for CC cells data points are mostly concentrated in the fourth quadrant (namely along positive *x* and negative *y*, which is the direction of the drift, see Fig. 1E); for CA cells data points are more homogeneous, but still there is a denser region corresponding to the first and the fourth quadrants, while for AA cells the distribution is homogeneous.

**Figure 4:**
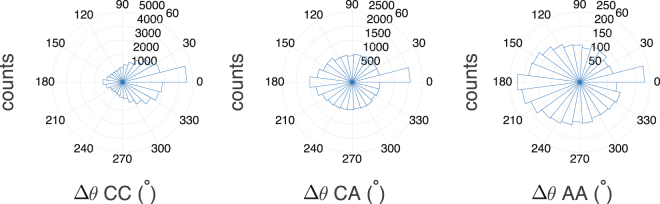
Polar histograms of the turning angle described by cells during their motion. For each cell we evaluate the turning angle Δ*θ* during the time lapse between two consecutive measurements and we analyze separately data pertaining to CC cells (left panel), to CA cell (central panel) and to AA cells (right panel). For each set, the raw values of the turning angle are grouped in bins, according to their numeric range. Notice that: in the CC case and in the CA case cells tend to maintain the same direction also if the frequency of steps preserving the direction of the motion is 2.5 times smaller for CA and the distribution is broader; in the AA case the distribution has almost zero mean and cells do not have a preferred direction.

Recalling the concepts introduced in^5^, we build the polar histogram of the turning angle ${\rm{\Delta }}\theta =\theta -\theta ^{\prime}$ (between two adjacent time lapses) along the whole trajectory in order to highlight any qualitative difference between the distributions pertaining to the three kind of cells considered. In this context a peaked histogram corresponds to a motion not prone to deviations, that is, to a focused trajectory that is pointing prevalently straight to its target, while a broader distribution corresponds to a relatively noisy and erratic trajectory, up to the limit of a uniform distribution that corresponds to an isotropic walk. The results collected are summarized in Fig. 4 and evidence smooth and very focused trajectories in the CC case, less smooth but rather focused in the CA case and just barely focused in the AA case.

Interestingly, these results show an analogous behavior respect to our previous work describing the immuno-competent vs immuno-deficient response in a murine model^5^, implying that this method can be considered a general tool for the immune system behavior analysis.

### Tumor-Immune cells interaction

#### Stability criteria for PBMCs approaching tumor cells

In the previous section we showed that AA cells have an extremely noisy behavior and CA cells perform sensibly worse than CC cells. We now restrict our study to CC immune cells only, looking at the stability of the convergence of PBMC trajectories, or, in other words, at the ability of PBMCs to maintain the focus towards their targets (TCs): crucially, in order to produce a true immunological defense, interactions between CCs and TCs must be stable^1^.

In the ideal and noiseless experiment, one could associate to each cell an equation of motion in the form $\dot{{\bf{x}}}=F({\bf{x}}(t))$, where ${\bf{x}}(t)$ is the vectorial position of the cell as a function of time and *F* is the driving force responsible for the motion, and derive the extent of the possible interaction between different cells. On the other hand, in the present case, nor we know the equation of motion for the single cell, neither this equation is expected to be deterministic as noise (stemming from several sources) comes into play. However, since we are not interested in the whole trajectory, but solely in its final stage, namely when the immune cell eventually collapses on the tumor cell, we can speculate on an heuristic similarity between the formal requirements of standard theoretical route in stability theory and the phenomenological behavior of our data in the neighborhood of TCs, assumed as fixed points. More in detail, in stability theory one usually looks for the fixed points **x**_0_ of the equation of motion (i.e., the points where $F({{\bf{x}}}_{0})=0$, namely where the velocity is zero), appointing them as *attractors*. Then, since for ${\bf{x}}\to {{\bf{x}}}_{0}$ the velocity is approaching zero, in the equation of motion one can retain solely the linear approximation of $F({\bf{x}}(t))$, that is $\dot{{\bf{x}}}={\mathbb{A}}{\bf{x}}$, where ${\mathbb{A}}\equiv \partial F/\partial {\bf{x}}{|}_{{\bf{x}}={{\bf{x}}}_{0}}$. The latter is the so-called Jacobian matrix and, in a 2-dimensional problem, ${\mathbb{A}}$ is a 2 × 2 matrix, which can be trivially diagonalized. Its eigenvalues ${\lambda }_{1},\,{\lambda }_{2}$ return crucial information regarding the stability of the *attractor* (e.g., the environment locally experienced by the migratory CCs)^23–25^:

- if both the eigenvalues are negative (i.e. ${\lambda }_{1}\,\le \,{\lambda }_{2}\, < \,0$), then the motion ends up on a stable fixed point;
- if both the eigenvalues are positive (i.e. ${\lambda }_{1}\,\ge \,{\lambda }_{2}\, > \,0$), then the motion ends up on an unstable fixed point;
- if ${\lambda }_{1}\, < \,0\, < \,{\lambda }_{2}$, then the candidate stationary point is a saddle-point and the motion is metastable.

**Figure 5:**
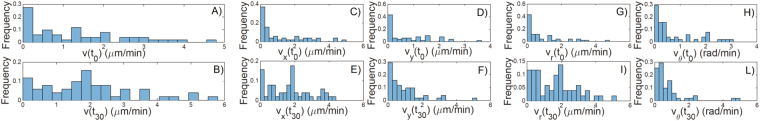
Histograms of the magnitude of the velocity and of its Cartesian and polar components for the PBMC distance from TC. For each TC shown in Fig. 1, we select the trajectories of the PBMC that reach a distance from TC shorter than the *physical interaction range*, evaluated as the sum of PBMC and TC mean diameters, and we calculate the instantaneous velocity $v(t)$ for all of them. The selected setting is novel but has been chosen in line with standard situations found in the literature. Panels A) and B) show the histograms of the magnitude of the velocity of each trajectory of the PBMC that move towards the selected TCs at the time step *t* = 0 (upper panel) and at the time step *t* = 30 (lower panel). As the trajectories are studied backward (such that when their collapse to the tumor cell, it is *t* = 0, while their initial positions, far from the tumor, belong to timeframe 30*s*), note that for *t* = 0 the mean velocity is peaked at 0, while for *t* = 30 the peak is shifted versus higher speeds (≈2.0 ± 0.8 μ m/min). Panels C)-F) show the histograms of the Cartesian components of the velocity at the time step *t* = 0 (upper panels) and at the time step *t* = 30 (lower panels). Note that, especially for *t* = 30, the distribution appears not symmetric, since ${v}_{y}$ does not change over time because the drift of PBMC motions towards the reservoir has a stronger bias along *x* rather than along *y*. Panels G)-L) show the histograms of the polar components of the velocity at the time step *t* = 0 (upper panels) and at the time step *t* = 30 (lower panels). Note that the angular velocity is always peaked at zero, as expected for quite smooth trajectories which point toward the tumor position, while the radial velocity is much spread at long times, when the interaction with the tumor is still weak. All the histograms are normalized by scaling between 0 and 1.

In the heuristic approach proposed here, we identify a TC with a candidate *attractor*. More in detail, taking into account that TCs positions remain roughly constant over time in the left chamber of the microfluidic platform described above (see Fig. 1), we can consider their time average (candidate) positions as fixed points for the PBMC dynamics. Hence, we identify the time average TC position with **x**_0_, since the instantaneous velocities of CC cells sensibly reduce when they approach the targeted tumor cells (see Fig. 5). More precisely, we need to relax the deterministic requirement $F({{\bf{x}}}_{0})=0$ toward $F({{\bf{x}}}_{0})\approx 0$ due to the presence of noise. This means that, for each PBMC, we effectively consider as fixed point the point of the trajectory that is closest to the TC position. Then, we estimate the four elements of the related Jacobian matrix ${\mathbb{A}}$ fitting the experimental data of $\dot{{\bf{x}}}$ versus **x** in the linear regime close to **x**_0_ for each PBMC approaching TC and we compute the matrix eigenvalues in order to infer the stability of the convergence of the PBMC to the TC (see Sections S2 and S3 for a detailed explanation). As a technical remark, we recall that, in general, cell tracks exhibit different spatial lengths because they may linger in the recorded window for a different amount of time. Also, when two particles have performed analogous distances from their initial point, their distance from the tumor cell can be very different. Hence, in order to properly compare different tracks, we shift the reference frame with respect to the time (and to the space), such that when (and where) the cells meet the TCs, we set that moment (and point) as the starting time of the analysis (and the origin of the reference frame). More precisely, for each track we set as time *t* = 0 the instant when the PBMC has its first interaction with TC. In this way, at the time *t* of any arbitrary track the PBMC is *t* steps far from the target, whatever loopy and noisy the trajectory may be. Similarly, for each track, we set as origin (*x* = 0, *y* = 0) the point occupied by the TC in such a way that the displacement $r=\sqrt{{x}^{2}+{y}^{2}}$ of a given PBMC in any arbitrary track means that the latter is at distance *r* from the tumor cell.

**Figure 6:**
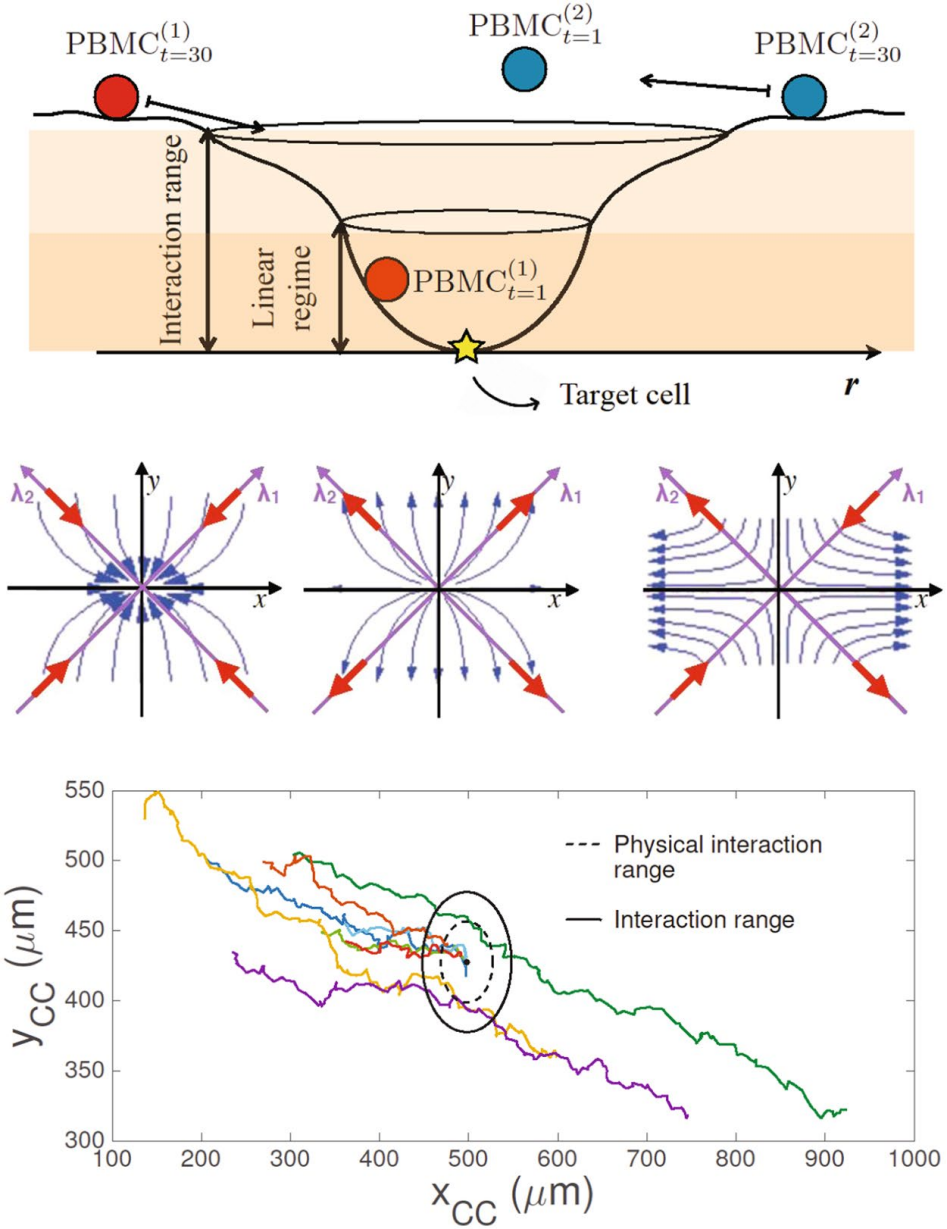
Upper panel: representative picture of the landscape generated by a tumor cell (i.e., the *attractor*) and possible dynamics of the immune cell. PBMC, attracted by the target TC, collapses in the minimum of the *linearization range* inside the *interaction range*. In the case of a stable trajectory (associated to the red sphere), any small perturbation at time 0 will not cause the escape of the particle from the minimum at time 1. On the contrary, metastable trajectories (associated to the blue sphere) are sensitive to small perturbations and the particle can easily escape from the local equilibrium. Central panel: plots of different possible immune cells dynamics. The panels resume the three different possible outcomes of the PBMC convergence to the TC *attractor* (stable, unstable and metastable dynamics), where the eigenvectors, labeled by λ, are the natural base built as a linear combination of λ_1_ and λ_2_. Bottom panel: real tracks of immune cells attracted by one tumor cell. The position of the tumor cell corresponds to the center of the black circle. The radius of the dashed circle represents the *physical interaction range* given by the sum of the mean diameter of PBMC (∼9 *μm*) and TC (∼20 *μm*). The radius of the solid circle represents the *interaction range* of the selected TC, (∼50 *μm*). The colored lines represent the tracks of the immune cells that approach the tumor cell considered. Each color corresponds to a different immune cell.

To complete our theoretical scenario, we consider that each tumor cell (i.e., each candidate *attractor* for PBMC) secretes diffusing chemoattractant (Annexin A1) which effectively generates a *local equilibrium* or *interaction range*: a PBMC that occurs to be within this range will eventually collapse on the related TC. Notice that the same TC can attract several PBMCs and the set of PBMCs that collapse on the same TC are said to belong to its related *basin* (see Fig. 6, top panel). Moreover, this *interaction range* is meant as the maximal distance where the PBMC under consideration may detect the TC’s chemoattractant gradient over the noise and, possibly, start to converge to the TC; clearly, this range is larger than the *physical interaction range*, that is the range where a physical interaction between the immune cells and the tumor cell occurs. The latter can be evaluated as the sum of the mean diameters of PBMC and TC (namely the time average value of the diameter of each TC and the time and ensemble average value of the diameters of all the PBMC). Within the *basin* of a single TC, we need also to define, for each PBMC, a *linearization range* as the range where the PBMC experimental data of $\dot{{\bf{x}}}$ versus **x** close to **x**_0_ follows a linear behavior (see Fig. 6 and Sec. 2.2 for a detailed explanation).

#### Stability of the convergence to tumor cells

In what follows, we present the application of the stability criteria described in the previous section to the case of one tumor cell that attracts a significant number of immune cells and we analyze solely the trajectories of those cells that reach a distance from the tumor cell equal to (or shorter) than the *physical interaction range* of the TC. Our refined dataset consists of 8 immune cells attracted by the tumor cell (see Fig. 6, bottom panel).

In particular, we explicit the analysis of the previous section calculating the Lyapunov coefficients λ_1_ and λ_2_ linked to two different experimental outcomes arising from the measured parameters of the PBMC trajectories: a stable and a metastable behavior in the PBMC approach to the TC (see Fig. 6, central panel); no evidence for a robustly unstable behavior is highlighted.

**Figure 7:**
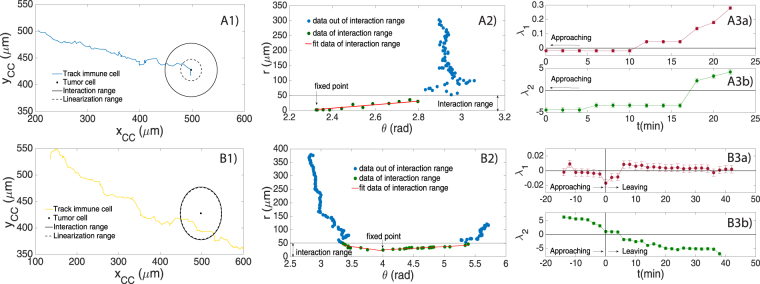
Analysis and comparison of the stable and metastable cases. In the left column (panels *A*1 and *B*1) bare Cartesian trajectories of PBMC are shown while moving towards the TC (black dot). The black continuous circle defines the *interaction range* while the inner dashed circle defines the *linearization range*. The *interaction range* has been defined considering the polar plots presented in the central column of the image (panels *A*2 and *B*2), which show, frame by frame, the distance *r* of the selected PBMC from the TC plotted as a function of the angle *θ*. Cell’s motion starts in the top right part of the plot (blue spots) and it is mainly noisy up to *r* ∼ 50 *μm* where a change in the direction is perceived, setting the boundaries of the *interaction range*. In this range (green spots) *r* decreases linearly with *θ* when the PBMC points towards the TC, and, only in the metastable case, it also increases linearly with *θ* when the PBMC leaves the TC after the interaction. In all the cases, the linear behaviors are fitted with the functions $r={p}_{1}\theta +{p}_{2}$ (red lines, panels *A*2 and *B*2), whose parameters are ${p}_{1}=\mathrm{62.67,}\,{p}_{2}=-144.61$ for the stable convergence (panel *A*2), ${p}_{1}=-\mathrm{31.17,}\,{p}_{2}=146.55$ in approaching the TC and ${p}_{1}=\mathrm{12.42,}\,{p}_{2}=-24.39$ in leaving the TC for the metastable convergence (panel *B*2). The *linearization range* is set by taking the PBMC experimental data of $({v}_{r},\,{v}_{\theta })$ versus (*r,θ*) close to (*r*_0_ *,θ*_0_) that show a linear behavior (see Figs S3 and S4). The linear regime is within ∼20 *μm* in the stable case and ∼49 *μm* in the metastable case. The right column (panels *A*3*a*,*b* and *B*3*a*,*b*) describes the temporal evolution of the Lyapunov coefficients λ_1_ and λ_2_ computed within the *linearization range*. Standard errors extracted from the best fits of the phase space analysis are calculated for each point. Notice that: in the stable case (*A*3*a*,*b*) for *t* → 0 they both converge to negative values thus confirming a stable match between PBMC and TC; in the metastable case (*B*3*a*,*b*) their values have almost always opposite sign (that they switch when they leave the fixed point) framing such a behavior as a saddle with regard to the prescribed TC as a candidate target cell.

In order to estimate the *interaction range* of the selected TC, we consider each PBMC trajectory in polar coordinates and we expect that, as each PBMC reaches the *basin’s* border, the fluctuations in the variable *θ* get softer and the slope *dr*/d*θ* should change abruptly (see panels *A*2 and *B*2 in Fig. 7), mirroring its fall toward the bottom of the local equilibrium. Within this range the target is engaged and we can check for a linear regime, in the approaching motion of the PBMC.

Next, we need to estimate a range where the PBMC experimental data of the velocities $(\dot{r},\,\dot{\theta })=({v}_{r},\,{v}_{\theta })$ versus (r,*θ*) close to (r_0_,*θ*_0_) follow a linear behavior. To this aim we define operatively a *linearization range* by retaining the largest number of data points (i.e., consecutive snapshots of the time lapse, starting from the one closer to the TC, hence the fixed point (r_0_,*θ*_0_) at *t* = 0), and going backward in time up to the borders of the *basin* such that, overall, the best linear fit of $({v}_{r},\,{v}_{\theta })$ versus (r,*θ*) displays a goodness coefficient lower bounded by *R* ^2^ = 0.95 (see Figs S3 and S4 in supplemental material).

Moreover, since we actually miss an explicit expression for the trajectory’s generator *F*((*r*,*θ*)(*t*)) and we therefore rely just on experimental data and operative definitions, we also accomplish a robustness check that consists in performing an extensive evaluation of the eigenvalues λ_1_ and λ_2_ over the approaching phase: rather than estimating the Jacobian spectrum just upon reaching *r*_0_ and inferring about stability at that point, we estimate the spectrum at all the time-points belonging to the *linearization range* in such a way that we get the time evolution of the Lyapunov coefficients too (see Fig. 7, panels *A*3 and *B*3). Indeed, in smooth enough scenarios, the evolution itself of λ_1,2_(*t*) should be consistent with the final values these eigenvalues assume (i.e., for (*r*,*θ*)→(*r*_0_,*θ*_0_))

The whole analysis is summarized in Fig. 7, where the two rows describe the stable and the metastable behavior, respectively, while the three columns show: the physical trajectory of the cell under study (left column); the plot, frame by frame, of the distance *r* of the PBMC from the TC as a function of the angle *θ* (central column); the obtained Lyapunov coefficients as functions of time (right column), calculated for the period during which the cells are inside the *linearization range* (dashed circle in the left panels).

**Figure 8:**
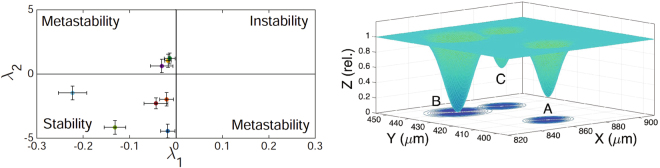
Left panel: plot of the Lyapunov coefficients (related to the immune cells attracted by the selected tumor cell). Left panel: each point (λ_1_, λ_2_) represents the Lyapunov coefficients extracted from the phase space analysis of each immune cells attracted by the tumor cell analyzed in this section. Both negative λ_1_ and λ_2_ identify the space of stability convergence, while both positive λ_1_ and λ_2_ identify the space of unstability convergence. When ${\lambda }_{1} < 0 < {\lambda }_{2}$, the candidate stationary point is in the metastable space. Standard errors extracted from the best fits of the phase space analysis are calculated for each point. Right panel: effective chemoattractant potentials generated by the tumor cells. Representative landscape of the chemoattractant potentials generated by three tumor cells as revealed by our approach. The minimum of each potential well corresponds to the position of a tumor cell in the physical space of the microfluidic chip (i.e., the plane of the spatial coordinates (x,y)). ${Z}_{(rel)}$ accounts for the depth of the potential well (normalized to the largest value), determined by its *local equilibrium* under the (standard) parabolic approximation: the larger the *local equilibrium*, the deeper the well. The *local equilibrium* is isolated when the *attractor* does not interact with the others (see potential well A). Conversely, when two *basins* partly overlap (see potential wells B and C), interference among their signal may result in metastability.

Focusing on the stable convergence to the *attractor* (Fig. 7, panels *A*1–*A*3), we observe the trajectory of the PBMC points towards the TC and, once the target is reached, the cell remains stack. During its movement, the PBMC starts to “feel” the attraction of the TC when it enters its *local equilibrium* (black circle in Fig. 7 panel *A*1), which is identified, from the numerical point of view, looking at the slope of *dr*/*dθ* in the polar plot (*r*,*θ*) (Fig. 7, panel *A*2): the point in correspondence of which, for decreasing $r$, $\theta$ stops to fluctuate and the slope *dr*/*dθ* changes beyond noisy fluctuations sets the boundary of the *interaction range* (threshold in panel *A*2). Finally, the *linearization range* (dashed black circle in Fig. 7, panel *A*1) is introduced as the range where it is possible to fit the velocity data $({v}_{r},\,{v}_{\theta })$ versus (*r*,*θ*) with a linear function (see Figs S3 and S4). Our analysis of velocity data in the linear regime shows that the radial velocity decreases linearly with *r* and *θ* reaching the value ${v}_{r}(t)\approx 0$ for $(r,\,\theta )\approx ({r}_{0},\,{\theta }_{0})$, where the TC is located: to be sharper, the uncertainty ${\rm{\Delta }}{v}_{r}$ we have on the radial velocity (introduced by the discrete time lapse) is larger then the actual velocity for *t* → 0, hence we can reasonably assume a negligible value for it. This suggests that the immune cell effectively approaches the tumor cell. On the contrary, the angular velocity increases linearly for decreasing $r\to {r}_{0}$ and $\theta \to {\theta }_{0}$ (i.e. approaching the TC) because the PBMC attacks the TC in different points, partially rotating around it (see Fig. S3). The parameters of this linear functions used to fit the velocity data are then used (see the mathematical details in supplemental material, Sec. S1) to empirically construct the ${\mathbb{A}}$ matrix, whose eigenvalues λ_1,_λ_2_, result to be both negative (see blue dot in Fig. 8), when computed in the fixed point where $(r,\,\theta )\approx ({r}_{0},\,{\theta }_{0})$, thus confirming the stable convergence of the PBMC to the TC. Moreover, the eigenvalues computed all over the *linearization range* become both negative in approaching the fixed point confirming the robustness of our approach (see Fig. 7, panel *A*3).

An example of a metastable convergence to the *attractor* is shown in Fig. 7, panel *B*1. The immune cell is initially attracted by the TC and deviates the trajectory. However, the cell does never reach a distance from the TC shorter than ∼25 *μm*, suggesting that no strong interaction occurs and the PBMC goes back to the original trajectory, moving away from the TC. Hence, in this case, we consider included in the *interaction range* the points of the immune cell trajectory both in approaching and in leaving the tumor cell. Such points are identified by the two linear fits shown in Fig. 7, panel *B*2, considering again the range where the slope *dr*/*dθ* changes abruptly. The related analysis of the velocity data (see Fig. S4) shows a parabolic behavior of both the angular velocity (convex parabola) and the radial velocity (concave parabola) respect to *θ* in proximity of the fixed point chosen (i.e. near the TC), due to the fact that the PBMC first approaches and, then, leaves the tumor cell. Moreover, the radial velocity and the angular velocity decrease and increase linearly with *r* respectively in approaching the TC, as detected in the stable case, again suggesting the existence of an interaction between the PBMC and the TC. However, such interaction seems to be weaker than in the stable case, considering that the immune cell does not overpass the boundaries of the *physical interaction range* and the radial velocity remains almost 1*μm*/*min*, also approaching the TC. Furthermore, the outcomes of the fits of the velocity data provide a positive and a negative value for the Lyapunov coefficients. This result allows us to identify the fixed point as a *saddle* thus determining the metastable convergence to the TC (see yellow dot in Fig. 8). A further confirmation is given by the analysis of the dynamics of the Lyapunov coefficients in the *linearization range*: for *t* → 0 (approaching the TC), λ_1_ becomes slightly negative while λ_2_ remains sensibly positive.

The analysis presented above was performed on the whole set of the immune cells attracted by the tumor cell shown in Fig. 6 (bottom panel). The outcomes are summarized in Fig. 8 (left panel), where we plot the Lyapunov coefficients obtained for each PBMC whose trajectory passes through the *physical interaction range*. Notice that, among the tracks analyzed, we have not found any robust evidence of unstable behavior (i.e., λ_1_, λ_2_ > 0). This may suggest that, once the PBMC enter the linearization range, the majority of the interactions are stable, confirming the presence of a robust immunological defence.

## Discussion

In this work we present a quantitative analysis of the migration of human PBMCs towards TCs and of the interactions among them, exploiting data collected by using a microfluidic platform and time lapse video analysis. We test our approach on a cell coculture where leukocytes, carrying or not a genetic polymorphism for FPR1, interact with human chemotherapy treated breast cancer cells. In order to characterize the dynamic of the system under study, we propose a set of numerical descriptors, some of which previously developed^5^. In particular, the analysis of step length distributions, velocities and turning angle of the moving PBMCs shows that leukocytes lacking the FPR1 gene or with only one copy of it (CA and AA) perform random walks without recognizing the chemotherapy-treated cancer cells or weakly pointing to them, while leukocytes with basal expression of FPR1 (CC) are able to perform random walks with drift toward the tumor cells and establish persistent interactions with them. In order to classify the stability of the trajectories, we, then, introduce a novel protocol based on Lyapunov stability criteria. This approach is encouraged by the fact that applications of stability criteria have been developed in a very broad setting^26^, even in the case of missing specific equations of motion or noisy environments^27^. In our case, TCs are considered as attractors and locally prevailing sources of the chemotactic gradients driving the PBMCs: while clearly we lack explicit equations of motion (hence, at a first glance, stability analysis would be hopeless), we know that white cell’s trajectories are highly not-uniform in their velocities: in particular, when far away, these cells run fast toward the target, then they slow down (approaching zero velocity) close to it, hence we are still allowed to check if, close to the candidate attractor (i.e. a tumor cell), locally their motion can be meaningfully tackled by a linear set of equations (that, ultimately, it is what is needed in order to perform stability analysis). We experimentally check that this is the case and we best-fit the linear dynamical system over the data-set to extrapolate its coefficients to be further analyzed: studying the eigenvalues of the Jacobian coupled to these coefficients, standard stability analysis can be performed, such that the signs of the Lyapunov coefficients in the plane of migration identify stable, metastable and (possibly) unstable trajectories.

In particular, our analysis does not detect the presence of any unstable case confirming the stable convergence of the immune cells towards the chemotherapy-treated cancer cells. Since such convergence is crucial for triggering an anticancer immune response, the numerical characterization could be useful to determine the beneficial effects of chemotherapy in the case under study. Nonetheless, at present, no protocols to inspect the probability of success of this event (i.e. a stable rather than an absent or transient interaction between PBMCs and TCs) are available. While a fully satisfactory approach is still out of reach, we believe that a semi-heuristic method is feasible and useful and we aim to propose the latter to overcome this flaw. In fact, microfluidic devices allow to fully exploit the modern microscopy tools and, through image data extraction, give access to the exploitation of the suitable mathematical tools able to quantify information. Certainly, the correspondence between the on-chip models with the *in-vivo* environment must be properly verified in each setting. In conclusion, our analysis clearly evidences the value of an integrated approach where OOC work perfectly as a bridge between biology and theoretical sciences for quantifying experiment outcomes and develop new theoretical models of the observed phenomena.

## Methods

### Experimental set up

The data-set analyzed in the present paper is based on data described in a recent paper by Vacchelli *et al*.^1^ that used the device shown in Fig. 1 to investigate the interactions between human PBMCs and doxorubicin treated human MDA-MB-231 breast cancer cells. PBMCs were obtained from healthy donors bearing the normal, most frequent FPR1 allele in homozygosis (CC), the RS867228 loss of function allele of FPR1 in heterozygosis (CA) and the RS867228 loss of function allele of FPR1 in homozygosis (AA).

### Video analysis and trajectory extraction

**Figure 9:**
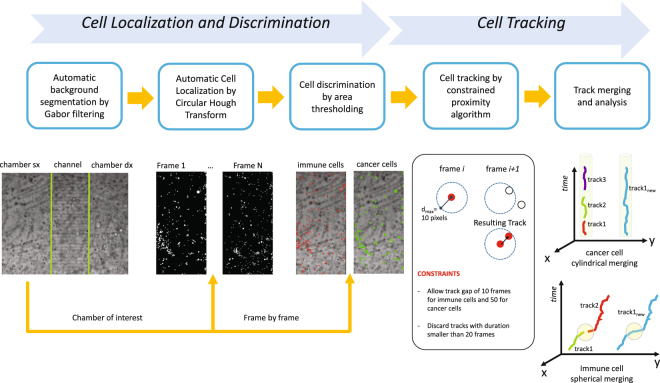
Schematic representation of the proposed tool. From left to right: background identification, cell localization, cell discrimination, individual cell tracking and track merging.

The cell cultures, described in detail elsewhere^1^, were observed by time lapse recordings by means of a Juli Smart microscope (Digital Bio) in a *CO*_2_ incubator at 37 °C. Microphotographs of a defined region of the device were taken at 2 minutes interval, with a 4*x* magnification objective. The obtained time lapse videos were then analyzed using a custom written software the architecture of which is schematized in Fig. 9.

The whole method can be divided into two sections: a first block devoted to the localization and, when needed, the discrimination of cells in the image and a second block committed to cell tracking. As pictured in Fig. 9, cell localization is structured into background analysis and cell location. The background is identified and the analysis is confined to the three distinct regions of the chip, which are automatically recognized: the left chamber, i.e. the chamber of interest in this example video as the location of immune-cancer interactions, a right chamber and the channel region.

After this step, cell location is performed by implementing a Circular Hough Transform (CHT)^28–30^ for each frame image in the video. PBMC and TCs discrimination is obtained by object area estimation implemented directly with the CHT approach. This procedure resulted as sufficiently reliable to discriminate the two populations, since the method attributes small objects to the immune cell class and largest objects to that of cancer cells, as shown in Fig. 9.

The second block in the proposed method implements cell tracking. According to the density and appearance of the cells, different approaches can be chosen for better tracking results^31^. In our application, the strong difference in dimension between immune and cancer cells as well as a not so high cell density in the image allowed to perform tracking using a Constrained Proximity Tracking (CPT) algorithm. Proximity tracking principle constructs a track by linking a cell in a frame *i* with the closest cell in the frame *i* + 1, within a maximum distance *d*_*max*_. However, due to the presence of cell populations with diversified motility characteristics, different distances have been considered: 10 pixels for immune cells (with high expected motility) and 5 pixels for cancer cells (with low expected motility). Of course, when no cells fall within the maximum distance from the given cell (missing tracking) or two different cells are going to be linked to the same cell (conflictual tracking), then the track stops. Track gaps have been also considered. Again due to cell-specific motility characteristics, a track gap of 10 frames for immune cells and of 50 frames for cancer cells is allowed.

The complexity of trajectories and the need to perform further analysis on them, require the application of constraints for extracting a subset of reliable tracks for the two populations. First, any track with duration smaller than 20 frames are eliminated since no further statistical analysis on motility can be performed on them. Second, due to the missing and the conflictual tracking situations, many short tracks are finally produced. The proposed approach performs a smart individual merging of tracks for the two populations. According to a low motility assumption for cancer cells, a cylindrical merging of tracks is iteratively performed on the tracks obtained for the cancer cell class. Conversely, due to the high motility of immune cells, a spherical local merging is iteratively applied to the tracks obtained for the immune cell class, as shown in Fig. 9.

The proposed method presents a novel approach to the problem of multi-population cell tracking. Simultaneous and independent cell localization and tracking is implemented for the first time for different cell populations, immune and cancer cells, using the cell discrimination step. Each individual cell tracking step has been compared with the standard software tool, TrackMate (a Fiji plugin^32^ for single particle-tracking)^33^. The results were congruent when compared using visual assessment, hence ready to further inspection.

## Electronic supplementary material

Supplementary Information
