# Supplementary material for: Organs on chip approach: a tool to evaluate cancer -immune cells interactions
Source: Sci Rep. 2017 Oct 6;7:12737. doi: 10.1038/s41598-017-13070-3 (PMC5630614; doi:10.1038/s41598-017-13070-3)
Supplement: Supplementary file 1 — Supplementary Information [file 41598_2017_13070_MOESM1_ESM.pdf]

Organs on chip approach: a tool to evaluate cancer - immune cells interactions

Elena Biselli[1], Elena Agliari[2], Adriano Barra[3], Francesca Romana Bertani[4], Annamaria Gerardino[4], Adele De Ninno[4,5], Arianna Mencattini[6], Davide Di Giuseppe[6], Fabrizio Mattei[7], Giovanna Schiavoni[7], Valeria Lucarini[7], Erika Vacchelli[8-12], Guido Kroemer[8-14], Corrado Di Natale[6], Eugenio Martinelli[6,*], and Luca Businaro[4]

[1]Department of Physics, Technische Universität München and Graduate School of Quantitative Biosciences, Ludwig-Maximilians-Universität München, James-Franck-Str. 1, 85748 Garching, Germany
[2]Dipartimento di Matematica, Sapienza Università di Roma, P.le A. Moro 2, 00185, Rome, Italy
[3]Dipartimento di Matematica e Fisica, Università del Salento, Via per Artesano, 73100, Lecce, Italy
[4]Institute for Photonics and Nanotechnologies, Italian National Research Council, Via Cineto Romano 42, 00156, Rome, Italy
[5]Department of Civil Engineering and Computer Science, University of Rome Tor Vergata, 00133 Rome, Italy
[6]Department of Electronic Engineering, University of Rome Tor Vergata, Via del Politecnico 00133, Rome, Italy
[7]Istituto Superiore di Sanità - Department of Oncology and Molecular Medicine, Viale Regina Elena 299, 00161, Rome, Italy
[8]Metabolomics and Cell Biology Platforms, Gustave Roussy Cancer Campus; Villejuif, France
[9]Université Paris Descartes, Sorbonne Paris Cité; Paris, France
[10]Equipe 11 labellisée Ligue Nationale contre le Cancer, Centre de Recherche des Cordeliers; Paris, France
[11]Institut National de la Santé et de la Recherche Médicale, U1138; Paris, France
[12]Université Pierre et Marie Curie, Paris, France
[13]Pôle de Biologie, Hôpital Européen Georges Pompidou, AP-HP; Paris, France
[14]Department of Women's and Children's Health, Karolinska University Hospital, 17176 Stockholm, Sweden
[*]martinelli@ing.uniroma2.it

Supplemental Material

1 Random walks and probability distributions

As anticipated in the main text, the analysis of the step length distributions along x and y axes, shown in Fig. 2, S1 and S2, allows us to identify which kind of random walk models at best the paths performed by leukocytes in the microfluidic device. Here we present the mathematical explanation.
We can model leukocytes' paths by means of random walks characterized by discrete time steps on a continuous two-dimensional space (xy). These random walks can be described in terms of a probability distribution $p(\mathbf{r},t)$ giving the probability that the walker has covered a distance $\mathbf{r}$ in a time t. In fact, we can write

$$p(\mathbf{r},t+\tau) = \int_{-\infty}^{\infty} p(\mathbf{r}',t)P(\mathbf{r}-\mathbf{r}',t)d\mathbf{r}', \tag{S1}$$

where $P(\mathbf{r}-\mathbf{r}',t)$ is the probability that at time t a step from $\mathbf{r}'$ to $\mathbf{r}$ is performed. For time-homogeneous processes, the width and the direction of a step do not depend on time and the dependence on t can be dropped, i.e. $P(\mathbf{r}-\mathbf{r}',t) = P(\mathbf{r}-\mathbf{r}')$. Moreover, by exploiting the discreteness of time steps, the time t at which any step occurs is a multiple of τ in such a way that we can write

$$p(\mathbf{r},n+1) = \int_{-\infty}^{\infty} p(\mathbf{r}',n)P(\mathbf{r}-\mathbf{r}')d\mathbf{r}', \tag{S2}$$

n being the number of steps performed up to the time considered and $t = \tau n$.

The distribution $P(\mathbf{r}-\mathbf{r}')$ qualitatively controls the resulting random walk, possibly giving rise to deterministic walks (e.g. $P(\mathbf{r}-\mathbf{r}') = \delta_{\mathbf{r}-\mathbf{r}',\mathbf{k}}$, $\mathbf{k} \neq \mathbf{0}$, corresponding to a ballistic motion) or to completely stochastic walks (e.g. $P(\mathbf{r}-\mathbf{r}') = \delta_{|\mathbf{r}-\mathbf{r}'|,\tilde{r}}$, corresponding to an isotropic motion where steps have fixed length $\tilde{r}$), etc[1].

In Euclidean structures, like the two-dimensional substrate considered here, we can decompose $\mathbf{r}$ into its normal coordinates, i.e. $\mathbf{r} = (x,y)$, and, analogously $\mathbf{r}-\mathbf{r}' = (x-x', y-y') \equiv (\Delta x, \Delta y)$. Therefore, Eq. S2 can be rewritten as

$$p((x,y),n+1) = \int_{-\infty}^{\infty}\int_{-\infty}^{\infty} p((x',y'),n)P(\Delta x,\Delta y)dx'dy', \tag{S3}$$

.

As suggested by Eq. S3, the knowledge of the specific distribution $P(\Delta x,\Delta y)$ possibly allows to get an explicit expression for $p((x,y),n)$.

For instance, one can show that, when diffusion is isotropic, i.e. it is equal in the x and y directions, any distribution $P(\Delta x,\Delta y)$ fulfilling the central limit theorem asymptotically (resuming the continuous time description) leads to the well-known diffusive limit characterized by the normal distribution

$$p((x,y),t) = \frac{1}{4\pi Dt} e^{-\frac{[(x-v_x t)^2+(y-v_y t)^2]}{4Dt}} \tag{S4}$$

where $\mathbf{v} = (v_x, v_y)$ accounts for the presence of a drift, while D is the diffusion-coefficient.

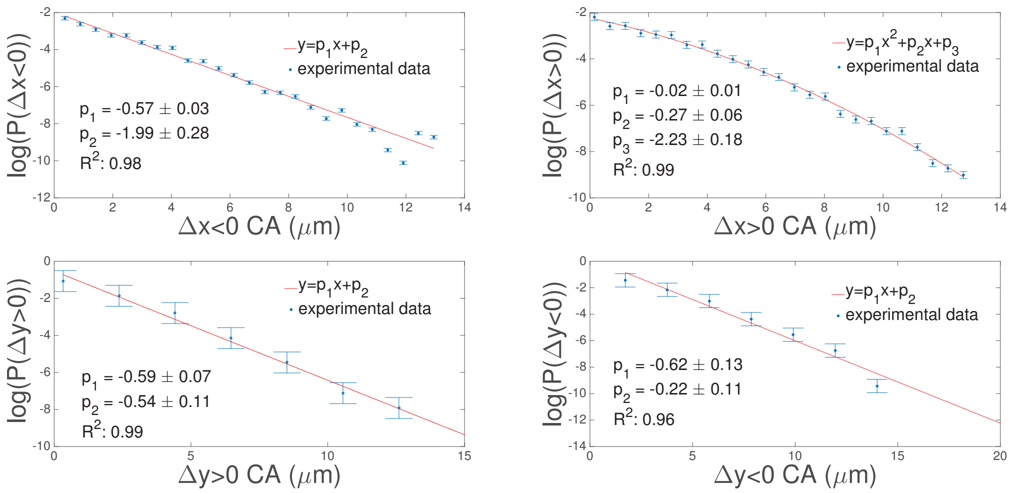

Figure S1. Step length distributions of CA cells. We repeat the analysis of Fig.1 focusing on CA cells. Experimental data with standard errors, fitting functions and the best-fit coefficients are reported in each panel. The distribution is exponential along negative x and Gaussian along positive x, as in the CC case. On the other hand, for the motion along the y direction, step lengths are distributed exponentially regardless of their sign. This suggests that the drift is weaker than the CC case and it acts mainly along the x direction.

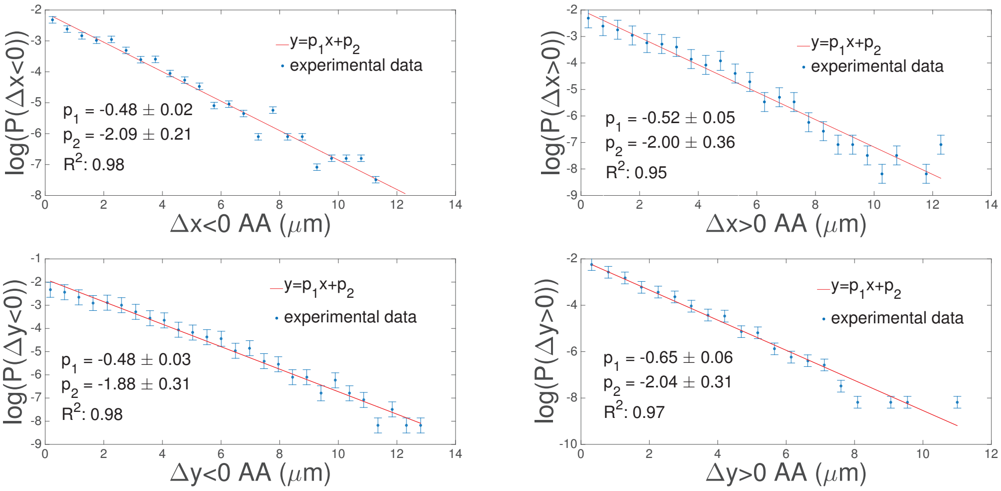

Figure S2. **Step length distributions of AA cells**. We repeat the analysis of Fig.1 focusing on AA cells. Experimental data with standard errors, fitting functions and the best-fit coefficients are reported in each panel. Along both x and y directions the distributions are exponential indicating that the motion displays no significant drift and the cells are expected to perform a random motion with no bias.

2 Application of Lyapunov stability criteria

We present a detailed mathematical explanation of our application of Lyapunov stability criteria used to analyze PBMCs behavior.

As anticipated in the main text (see Sec. 2.1), we associate to each PBMC an equation of motion in the form $\dot{\mathbf{x}} = F(\mathbf{x}(t))$, where $\mathbf{x}(t)$ is the vectorial position of the cell as a function of time and F is the driving force responsible for the motion. Then, we consider each TC position as a fixed point $\mathbf{x}_0$ of the equation of motion (i.e., the points where $F(\mathbf{x}_0) = 0$, namely where the velocity is zero), appointing them as candidate *attractors*. A key remark to support this point is that, as expected from stability theory, the velocities of CC cells sensibly reduce when they approach the targeted TC (see Fig. 5). However, we need to relax the deterministic requirement $F(\mathbf{x}_0) = 0$ toward $F(\mathbf{x}_0) \approx 0$ due to the presence of noise. This means that, for each PBMC trajectory, we effectively consider as a fixed point the one that is closest to the TC position. Then, in order to characterize this as a fixed point, we need to estimate the four entries of the related Jacobian matrix $\mathbb{A}$. To this aim we can not rely on analytical methods because, as underlined in the main text, we do not have the explicit expression for $F(\mathbf{x})$. On the other hand, we have experimental data for $\dot{\mathbf{x}}$ versus $\mathbf{x}$ and we can directly fit them, as long as the linear condition is verified at least in the spatial region close to $\mathbf{x}_0$.

Following this route we study in details each trajectory recorded for CC cells approaching a TC, namely the evolution of the position $\mathbf{x}$ traced by each single CC cell in the neighborhood of $\mathbf{x}_0$. This analysis is accomplished in the phase space $(\dot{\mathbf{x}}, \mathbf{x})$, where $\dot{\mathbf{x}}$ is the velocity of the cell, calculated in either Cartesian (data not shown) or polar coordinates, while $\mathbf{x}$ is the position of the cell, calculated in either Cartesian (data not shown) or polar coordinates.
Assuming a smooth motion close to TC, we can effectively quantify CC's behavior close to the TC (i.e. for $t = 1, 2, \ldots$) by fitting the data points of $\dot{\mathbf{x}}$ versus $\mathbf{x}$ seeking for a linear relation that mathematically can be represented as

$$v_x = \dot{x} = A_{x,x}x + A_{x,y}y, \tag{S5}$$
$$v_y = \dot{y} = A_{y,x}x + A_{y,y}y, \tag{S6}$$

for Cartesian coordinates, or as

$$v_r = \dot{r} = A_{r,r}r + A_{r,\theta}\theta, \tag{S7}$$
$$v_\theta = \dot{\theta} = A_{\theta,r}r + A_{\theta,\theta}\theta, \tag{S8}$$

in polar coordinates.
Focusing on the latter case, we can recast the related equations compactly exploiting a matricial notation as

$$\begin{array}{l} \dot{\mathbf{r}} = (v_r, v_\theta)^T, \mathbf{r} = (r, \theta)^T \\ \mathbb{A} = \begin{pmatrix} A_{r,r} & A_{r,\theta} \\ A_{\theta,r} & A_{\theta,\theta} \end{pmatrix} \end{array} \Rightarrow \dot{\mathbf{r}} = \mathbb{A}\mathbf{r}. \tag{S9}$$

3 Calculus of the Jacobian matrix elements

In the heuristic approach proposed and applied in the main text (see Sec. 2.1 and 2.2) we identify a TC with a candidate *attractor*, that is, we identify the TC position with $\mathbf{x}_0$, we estimate the four elements of the related Jacobian matrix $\mathbb{A}$ fitting the experimental data of $\dot{\mathbf{x}}$ versus $\mathbf{x}$ in the linear regime close to $\mathbf{x}_0$ for each PBMC approaching TC and we compute the matrix eigenvalues in order to infer the stability of the convergence of the PBMC to the TC. In polar coordinates (r, θ) this means that we fit the experimental data of (v_r, v_θ) versus (r, θ) in the linear regime close to (r_0, θ_0) for each PBMC approaching TC.

Here we present the fitting analysis of the velocity data for the stable and metastable case shown in Sec. 2.2 from which we can extract the elements of the Jacobian matrix and the related Lyapunov coefficients.

In each case we plot the polar components of the velocity v_r and v_θ of the immune cell versus the polar coordinates r and θ within the *linearization range*.

In the stable case, the velocities show a linear behavior up to a distance of $\sim 20\mu m$ from the TC (see Fig. S3). As pointed out in the main text (see Sec. 2.2), the radial velocity decreases linearly with r and θ, while the angular velocity increases linearly for decreasing $r \to 0$ and $\theta \to \theta_0$. The fitting functions in this *linearization range* are $\mathbf{y} = p_1\mathbf{x} + p_2$, where $\mathbf{y} = (v_r, v_\theta)$, $\mathbf{x} = (r, \theta)$ and p_1 are the elements of the Jacobian matrix. From the diagonalization of this matrix we obtain two negative eigenvalues $(\lambda_1, \lambda_2) = (-0.02 \pm 0.01, -4.45 \pm 0.01)$ that confirm the stable convergence of the trajectory to the fixed point (r_0, θ_0) (see blue dot in Fig. 8, left panel).

In the metastable case, the velocities show a linear behavior up to a distance of $\sim 49\mu m$ from the TC (see Fig. S4). As shown in the main text (see Sec. 2.2), radial and angular velocities decrease and increase linearly with r respectively, while they present a parabolic behavior respect to θ in proximity of the fixed point chosen (i.e. near the TC), due to the fact that the PBMC first approaches and, then, leaves the tumor cell. In the first two cases (Fig. S4, upper panels), the fitting functions are $\mathbf{y} = p_1\mathbf{x} + p_2$, where $\mathbf{y} = (v_r, v_\theta)$, $\mathbf{x} = (r, \theta)$ and p_1 are two elements of the Jacobian matrix. In the case of the parabolic behavior, we linearize the fitting function $\mathbf{y} = p_1\mathbf{x}^2 + p_2\mathbf{x} + p_3$ around the fixed point $\mathbf{x_0}$ where $F(\mathbf{x_0}) \approx 0$, obtaining the other two elements of the Jacobian matrix, whose diagonalization gives the positive and negative eigenvalues $(\lambda_1, \lambda_2) = (-0.02 \pm 0.01, 1.04 \pm 0.01)$ that confirm the metastable convergence of the trajectory to the fixed point (r_0, θ_0) (see yellow dot in Fig. 8, left panel).

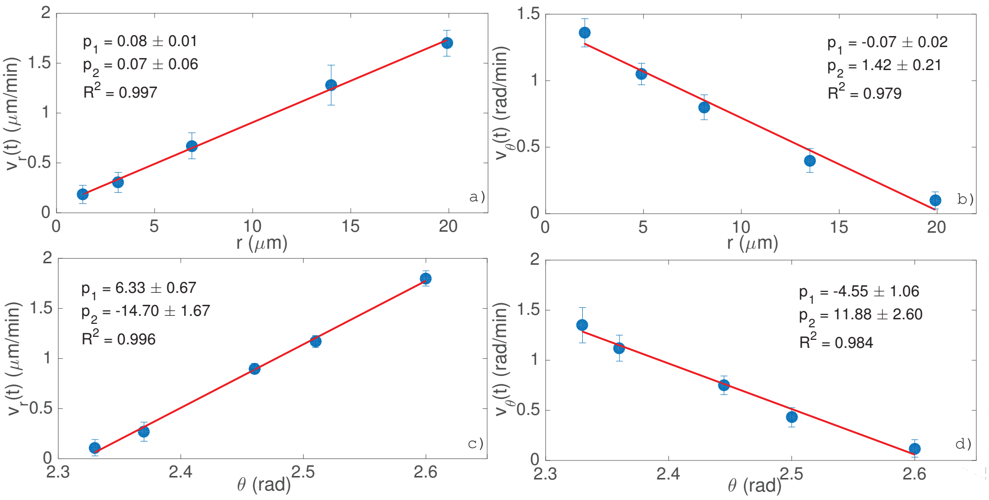

Figure S3. Analysis of the stable case. The polar components of the velocity v_r and v_θ of the immune cell showing stable convergence (see Fig. 7, panels $A1-A3$) are plotted versus the polar coordinates r and θ within the *linearization range*. The fitting function is $\mathbf{y} = p_1\mathbf{x} + p_2$ and the best-fit coefficients are reported for each panel, where $\mathbf{y} = (v_r, v_\theta)$, $\mathbf{x} = (r, \theta)$ and p_1 are the elements of the Jacobian matrix. The resulting R^2 of the fits are, respectively, $R^2_{v_r,r} = 0.997$, $R^2_{v_\theta,r} = 0.979$, $R^2_{v_r,\theta} = 0.996$, $R^2_{v_\theta,\theta} = 0.984$ and confirm the locally linear behavior of the PBMC trajectory in proximity of the TC (*attractor* point). Experimental data with standard errors are always grouped in bins with the same number of points in each bin.

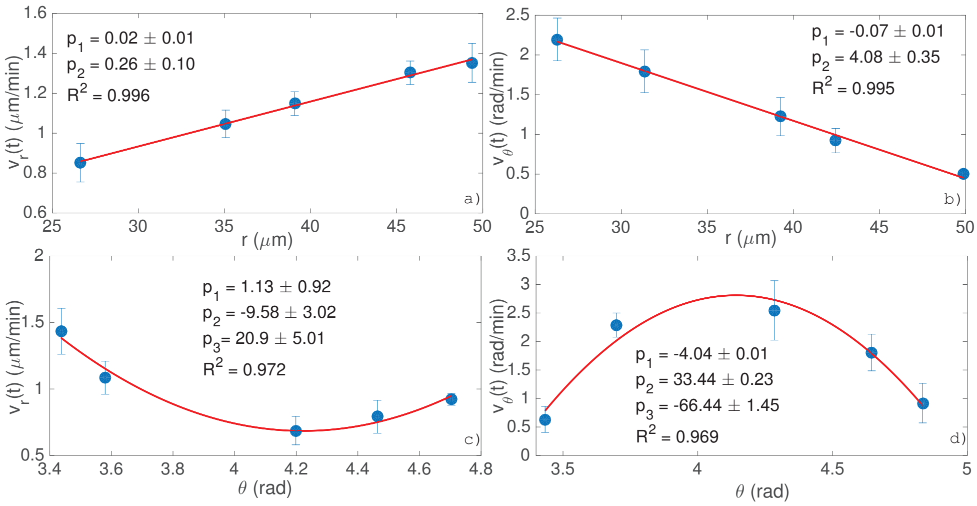

Figure S4. Analysis of the metastable case. The polar components of the velocity v_r and v_θ of the immune cell showing metastable convergence (see Fig. 7, panels $B1-B3$) are plotted versus the polar coordinates r and θ within the *linearization range*. The fitting functions are $\mathbf{y} = p_1\mathbf{x} + p_2$ (linear fit) and $\mathbf{y} = p_1\mathbf{x}^2 + p_2\mathbf{x} + p_3$ (quadratic fit) and the best-fit coefficients are reported for each panel, where p_1 of the linear fit and $2p_1\mathbf{x_0} + p_2$ (with $F(\mathbf{x_0}) \approx 0$) obtained from the linearized quadratic fit are the elements of the Jacobian matrix. The resulting R^2 are, respectively, $R^2_{v_r,r} = 0.996$, $R^2_{v_\theta,r} = 0.995$, $R^2_{v_r,\theta} = 0.972$, $R^2_{v_\theta,\theta} = 0.969$ and confirm the locally linear behavior of the PBMC trajectory in proximity of the TC (*attractor* point). Experimental data with standard errors are always grouped in bins with the same number of points in each bin.

References

1. Weiss, G.H., *Aspects and applications of random walks*, North-Holl. Press, Amsterdam. (1994).
